# Human milk oligosaccharides, antimicrobial drugs, and the gut microbiota of term neonates: observations from the KOALA birth cohort study

**DOI:** 10.1080/19490976.2022.2164152

**Published:** 2023-01-08

**Authors:** D.J.M Barnett, M.F Endika, C.E Klostermann, F Gu, C Thijs, A Nauta, H.A Schols, H Smidt, I.C.W Arts, J Penders

**Affiliations:** a Maastricht Centre for Systems Biology (MaCSBio), Maastricht University, Maastricht, The Netherlands; b Department of Medical Microbiology, Maastricht University Medical Center, Maastricht, The Netherlands; c Laboratory of Microbiology, Wageningen University & Research, Wageningen, The Netherlands; d Biobased Chemistry and Technology, Wageningen University & Research, Wageningen, the Netherlands; e Laboratory of Food Chemistry, Wageningen University & Research, Wageningen, The Netherlands; f Department of Epidemiology, Maastricht University, Maastricht, The Netherlands; g CAPHRI Care and Public Health Research Institute, Maastricht University Medical Center, Maastricht, The Netherlands; h FrieslandCampina, LE Amersfoort, The Netherlands; i NUTRIM School of Nutrition and Translational Research in Metabolism, Maastricht University Medical Center, Maastricht, The Netherlands

**Keywords:** Infant nutrition, human milk oligosaccharides, antibiotics, gut microbiome, breastfeeding, prebiotics

## Abstract

The infant gut microbiota affects childhood health. This pioneer microbiota may be vulnerable to antibiotic exposures, but could be supported by prebiotic oligosaccharides found in breast milk and some infant formulas. We sought to characterize the effects of several exposures on the neonatal gut microbiota, including human milk oligosaccharides (HMOs), galacto-oligosaccharides (GOS), and infant/maternal antimicrobial exposures. We profiled the stool microbiota of 1023 one-month-old infants from the KOALA Birth Cohort using 16S rRNA gene amplicon sequencing. We quantified 15 HMOs in breast milk from the mothers of 220 infants, using high-performance liquid chromatography-mass spectrometry. Both breastfeeding and antibiotic exposure decreased gut microbial diversity, but each was associated with contrasting shifts in microbiota composition. Other factors associated with microbiota composition included C-section, homebirth, siblings, and exposure to animals. Neither infant exposure to oral antifungals nor maternal exposure to antibiotics during pregnancy were associated with infant microbiota composition. Four distinct groups of breast milk HMO compositions were evident, corresponding to maternal Secretor status and Lewis group combinations defined by the presence/absence of certain fucosylated HMOs. However, we found the strongest evidence for microbiota associations between two non-fucosylated HMOs: 6’-sialyllactose (6’-SL) and lacto-N-hexaose (LNH), which were associated with lower and higher relative abundances of *Bifidobacterium*, respectively. Among 111 exclusively formula-fed infants, the GOS-supplemented formula was associated with a lower relative abundance of *Clostridium perfringens*. In conclusion, the gut microbiota is sensitive to some prebiotic and antibiotic exposures during early infancy and understanding their effects could inform future strategies for safeguarding a health-promoting infant gut microbiota.

## Introduction

The infant gut microbiota is important for childhood health. It acts both as a guiding force for balanced immune system development^1,2^ and as the starting point for ecological succession toward a stable adult-like microbiota, with lifelong importance for metabolic, gastrointestinal, and even neuropsychological health.^3,4^ Thus, it is critical to nurture this ecosystem as it establishes and to avoid or mitigate harmful perturbations.

Antimicrobial agents could be an important cause of such perturbations in some children. Direct antibiotic exposures have been shown to cause long-lasting collateral damage to the commensal gut microbiota in infants and children.^5–7^ Recent work by van Tilburg Bernardes et al.^8^ also suggests a role for fungal commensals in neonatal microbiome development, and implies that disturbance of the mycobiome could have consequences for the bacterial microbiota. Accordingly, another *in vivo* study showed that the antifungal fluconazole affected the bacterial microbiota as well as host immune responses.^9^ Furthermore, indirect exposure to antimicrobial agents might also affect infant microbiota establishment. Antibiotics taken by pregnant women have been associated with increased risk of immune-mediated and metabolic diseases in their children, such as asthma and obesity, leading some to speculate that these associations could be mediated by effects on the infant microbiota that follow perturbations of the mother’s microbiome.^10–16^

The importance of safeguarding infant microbiota development is underscored by human evolution: mothers produce a large variety of digestion-resistant carbohydrates in breastmilk, namely human milk oligosaccharides (HMOs), primarily for the benefit of the infant’s microbiota. Today’s infant formulas also often include some simple oligosaccharide structures, with the aim of conveying similar microbiota-targeted benefits. Galacto-oligosaccharides (GOS) were one of the earliest of these additions, followed by the HMO 2’-fucosyllactose (2’-FL). Both have shown microbiota effects and health benefits in some preclinical and clinical studies.^17,18^ However, breast milk provides a much more complex mixture of HMOs: over 150 structures have been elucidated, the vast majority of which are understudied, and hundreds more are detectable in small quantities.^19,20^ Breastmilk HMO composition can vary greatly between mothers, due in large part to variations in the Secretor (*FUT2*) and Lewis (*FUT3*) genes. A few small cohort studies have examined associations between breastmilk HMO concentrations and the infant microbiota, but their conclusions are mixed, and limited by the highly imbalanced population distribution of the Secretor and Lewis groups.^21–26^ For example, Laursen et al. recently found no major effects of maternal Secretor status on the infant microbiota, but only five of the 34 mothers included were non-Secretors (inactive *FUT2*), and Lewis status was not considered.^24^

We need to refine our understanding of how infant microbiota composition and assembly can be influenced by factors such as these naturally varied breast milk HMOs, because this could inform future strategies to support healthy microbiota development and to mitigate collateral damage from antimicrobial exposures. Using data from 1023 infants in the KOALA birth cohort, we examined the effects of pre- and post-natal antibiotic and antifungal exposures on the microbiota of term neonates. Moreover, we characterized natural variation in the concentrations of 15 abundant HMOs, measured in the breast milk samples of 220 mothers, and examined their associations with the gut microbiota of breastfed neonates. Additionally, in 111 exclusively formula-fed neonates, we explored gut microbiota differences associated with the use of GOS-supplemented infant formula.

## Results

### Study population

**Figure 1.**
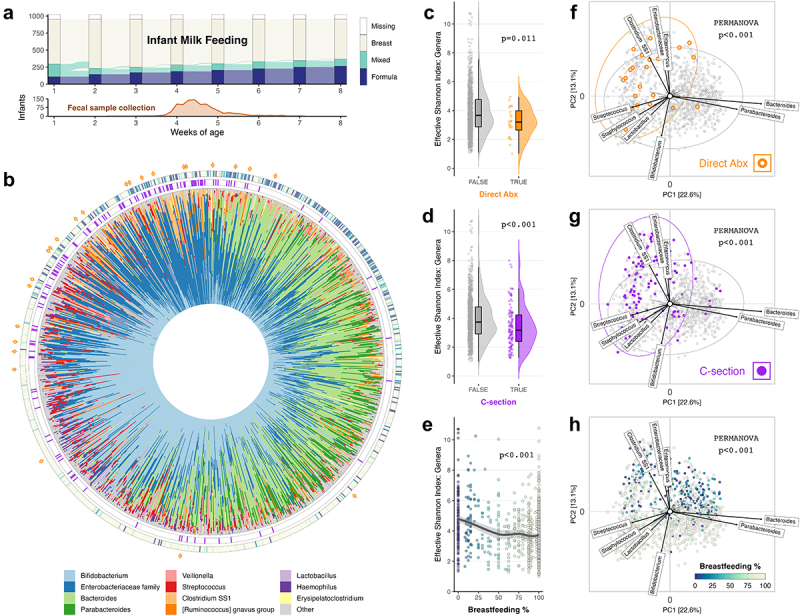
Microbiota composition at 1 month related strongly to antibiotics, C-section and breastfeeding history (N = 1023).

In total, 1023 infants were included in our analyses (Supplementary Table S1), approximately 66.5% (680/1023) of whom were breastfed from birth until age of fecal sample collection, at a median of 31 days (IQR 29–34, Figure 1(a)). Almost half of the 237 infants that were formula-fed at sample collection were exclusively formula-fed from birth onwards (111/237). Approximately 2.2% of the infants (23/1023) received antibiotics (Supplementary Table S2) prior to fecal sample collection, 3.6% (37/1023) of infants received oral antifungals, 15.2% of the mothers reported antibiotic use during pregnancy (155/1023), and 11.1% of the infants were born by C-section (114/1023). Overall, 31.6% of the mothers (323/1023) were enrolled via the “alternative” recruitment strategy (see Methods), which correlated positively with breastfeeding, maternal age at delivery, and educational level (Supplementary Figure 1).

(a) Alluvial diagram indicating the number of children in each feeding category at each week, and the number of infants transitioning between groups each week. Frequency polygon indicating the distribution of ages at which infant fecal samples were collected (in days). (b) Circular stacked bar chart showing the genus-level taxonomic composition of each sample as relative abundances. Samples are arranged in the same order as in PCA plots (f-h) by their anticlockwise rotational order around the origin, starting from above the x-axis in the upper-right quadrant. Each sample is annotated with C-section (purple), breastfeeding (color gradient), and direct antibiotic (orange) information in concentric rings from the inside outwards. (c-d) Raincloud plots showing the distribution of Effective Shannon index at genus level, grouped by antibiotic exposure (c) and C-section (d), and p-values for association from covariate-adjusted linear regression (Supplementary Table S3). (e) Scatterplot of Effective Shannon index at genus level in association with breastfeeding as a proportion of total milk feeding prior to fecal sample collection, and p-value from covariate-adjusted linear regression (Supplementary Table S3). A simple LOESS regression line of the best fit is overlaid with 95% confidence interval. (f-h) PCA biplots using CLR-transformed genus-level microbial features. Taxon loading vectors labeled for the taxa that contributed most to microbiota variation along each PC axis. Plots are colored by direct antibiotic (Abx) exposure (f), C-section birth (g), and breastfeeding proportion (h), and the p-values shown are for the association of each exposure with microbiota composition, from covariate-adjusted PERMANOVA (Supplementary table S4). The 95% confidence ellipses are added to plots f & g. Percentages at the axes indicate the amount of variation explained.

### Breastfeeding, birth mode, and direct antibiotic exposure shape the one-month microbiota

#### Microbiota diversity

We identified 213 genus-level taxa across all infants, but the microbiota of most infants was predominated by taxa from only a few genera, including *Bifidobacterium, Bacteroides*, and genera from the *Enterobacteriaceae* family (Figure 1(a)). Most of the remaining genera were rare, with only 39 genera detected in at least 2.5% of the infants, and a median of 11 genera per infant (IQR 9–13). Direct antibiotic exposure (β = −0.78, 95% CI [−1.39, −0.18]), C-section birth (−0.61 [−0.91, −0.30]), and a higher proportion of breastfeeding (−1.04, [−1.32, −0.77]) were all statistically significantly associated with a decreased microbiota diversity at 1 month (Figures 1(c-e), Supplementary Table S3).

#### Microbiota composition

While antibiotics, C-section and breastfeeding were all related to reduced microbial diversity, each had a different pattern of association with microbiota composition (Figure 1(f-h)). Multivariable PERMANOVA analyses revealed that these factors were significantly independently associated with microbiota composition (Aitchison distance on genera, p < .001, Supplementary Table S4). Several other exposures were also significantly associated (p < .05) with microbiota composition including homebirth; presence of older siblings; exposure to furry pets or farm animals; and age of fecal sample collection. In contrast, we did not find significant associations between infant gut microbiota composition and maternal use of antibiotics during pregnancy, either during the third trimester or at any time, despite observing 155 prenatal exposures. Additionally, we did not identify any evidence to suggest that infant microbiota composition was associated with either neonatal exposure to oral antifungals or maternal use of antibiotics whilst breastfeeding. Models using alternative distance measures, Bray-Curtis and Generalized-UniFrac, produced comparable results (data not shown).

**Figure 2.**
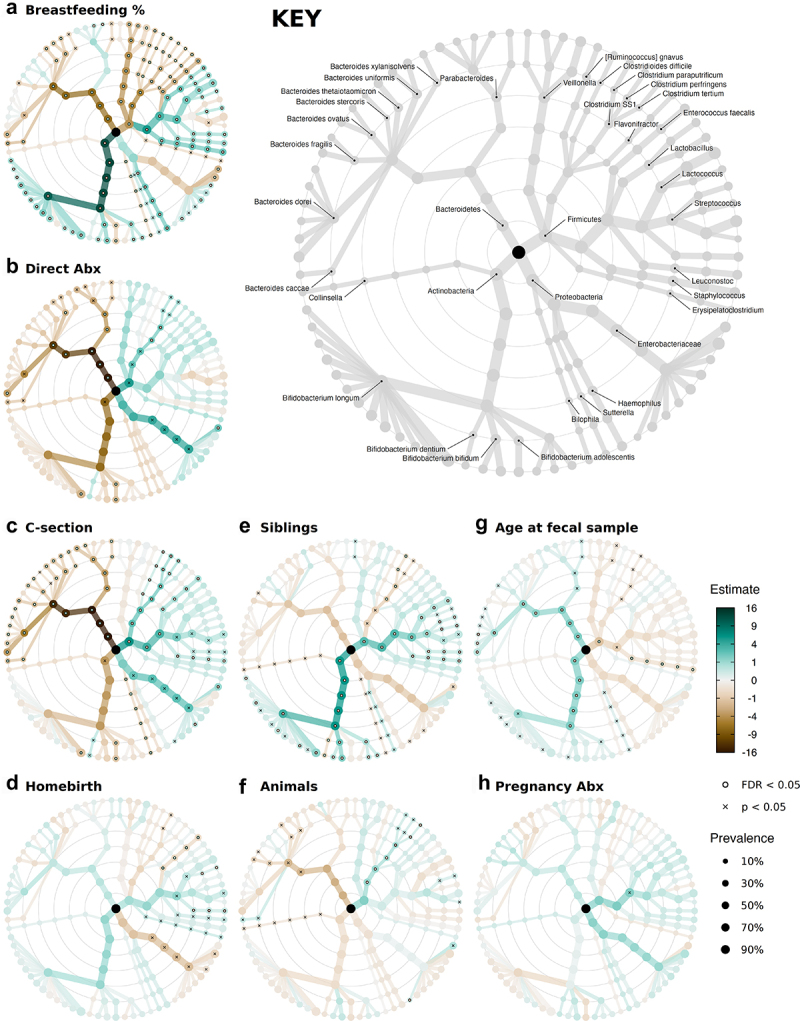
Several exposures associated independently with gut microbiota composition.

The proportion of breastfeeding received explained the largest variation in microbiota composition in PERMANOVA analyses (Supplementary Table S4); infants that received more breastfeeding typically had a substantially different microbial composition to infants more often fed formula (Figure 2(a)). Breastfeeding was associated with the majority of taxa, and notable associations (FDR < .05) included significantly higher relative abundances of the genus *Bifidobacterium* and most members of the class *Bacilli*, including the lactose fermenters *Lactobacillus, Lactococcus*, and the skin-resident facultative anaerobe *Staphylococcus*, as well as significantly lower relative abundances of the genera *Bacteroides* and *Veillonella*, and the class *Clostridia*.

(a-h) Taxonomic association trees: visual summary of the results of covariate-adjusted regression models of log2-transformed relative abundances, showing the strength, direction, and statistical significance of the independent metadata-to-microbe associations modeled at all ranks from Phylum (innermost ring) to ASV (outermost ring). The color intensity of each node represents the regression coefficients for the associations between the exposure variable and a microbial taxon. Absolute values greater than 16 are colored as ±16. The node size indicates the prevalence of that taxon, and only taxa with prevalence of at least 5% were included in these analyses. Nodes overlaid with a white dot represent statistically significant associations after BH correction, FDR < .05 per rank. Crosses indicate unadjusted p < .05. KEY indicates which microbial taxon is represented at each node. The full results are presented in Supplementary Table S5. All models were adjusted for the same covariates as in the PERMANOVA and alpha-diversity regression models (see Methods).

We observed a different pattern of strong microbial associations in the 23 infants directly exposed to antibiotics (Figure 2(b)). *Clostridium sensu stricto* 1 (*Clostridium* SS1) was significantly more abundant on average in antibiotic-exposed infants (FDR = .037), and we observed large associations with higher relative abundances of taxa from the *Enterobacteriaceae* family (ASV2, FDR = .034). Antibiotic exposure is associated with lower *Bacteroides* (FDR < .001) and *Parabacteroides* (FDR = .024) relative abundances, in particular with significant depletion of *B. caccae, B. uniformis, B. dorei, P. distasonis*, and *P. merdae* at the species level (p < .05). A large decrease in the relative abundance of *Bifidobacterium* was also observed, although this only reached statistical significance for a few ASVs within the species *B. longum* and *B. adolescentis* (p < .05).

Similar to direct antibiotic exposure, C-section birth showed large and significant independent associations (Figure 2(c)) with a decreased relative abundance of the *Bacteroidetes* phylum, including *Bacteroides* and *Parabacteroides* (FDR < .001) and with a greater relative abundance of *Clostridium* SS1 (FDR < .001), as well as increases in taxa in the *Enterobacteriaceae* family (FDR = .07). Conversely, being born at home was significantly associated with a lower relative abundance of *Enterobacteriaceae*, relative to hospital-born infants (Figure 2(d)).

Additionally, the presence of older siblings was associated with an increased relative abundance of *Bifidobacterium* as well as *Streptococcus* and *Lactococcus* and a lower *Clostridium* SS1 relative abundance (FDR < .01, Figure 2(e)), and neonatal animal exposure was weakly associated with lower *Bacteroides* and higher *Clostridium* SS1 relative abundance (p < .05, Figure 2(f)). Fecal samples collected at later ages had significantly higher relative abundances of *Bacteroides* and *Bifidobacterium* (FDR < .05, Figure 2(g)), despite the narrow age distribution (SD 4 days).

### Natural variation in breastmilk HMO concentrations shows associations with microbiota

**Figure 3.**
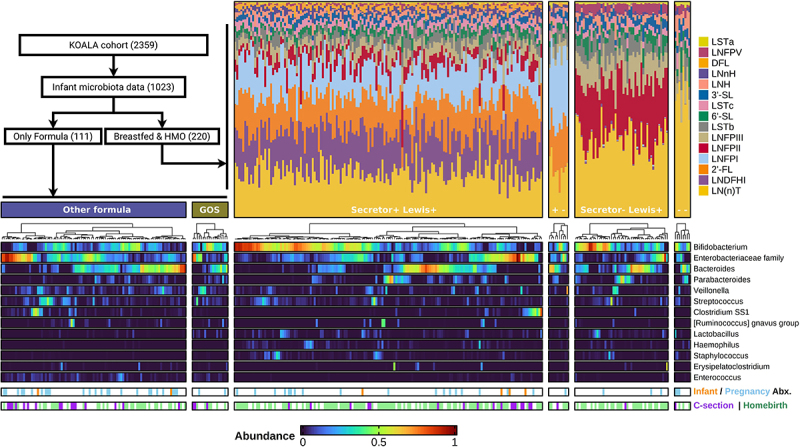
Annotated heatmaps of microbial composition showed high interindividual variation within feeding-type groups.

Even with our detailed information on multiple exposures strongly associated with the infant microbiota, such as the weekly breastfeeding history, most variations in microbiota composition at 1 month remained unexplained (Supplementary Table S4). Aiming to explain some part of this remaining microbial variation, we examined further details of infant nutritional intake using two subgroups of the study population: exclusively breastfed infants, and exclusively formula-fed infants (Figure 3). First, we explored the natural variation in breast milk HMO profiles from mothers of 220 exclusively breastfed infants, and assessed associations of 15 HMOs with infant gut microbiota composition.

Inset flowchart indicates the source of 111 formula-fed children and 220 exclusively breastfed children with maternal HMO data. Annotated heatmaps of infant fecal microbiota relative abundance grouped by feeding-type: GOS-supplemented and other formula-fed infants and breastfed infants grouped by maternal Secretor/Lewis status. The top 13 genus-level taxa are displayed in order of mean total counts across the study population, and infants in each heatmap are sorted by microbial composition similarity using hierarchical clustering. Breastfed infants are annotated with breast milk HMO compositions as stacked bar charts of proportions. The two lower annotation strips indicate infant antibiotic exposure and maternal antibiotic exposure during pregnancy for each mother-child dyad, as well as C-section birth, homebirth, or hospital vaginal delivery (white).

#### Breastmilk HMO concentrations

**Figure 4.**
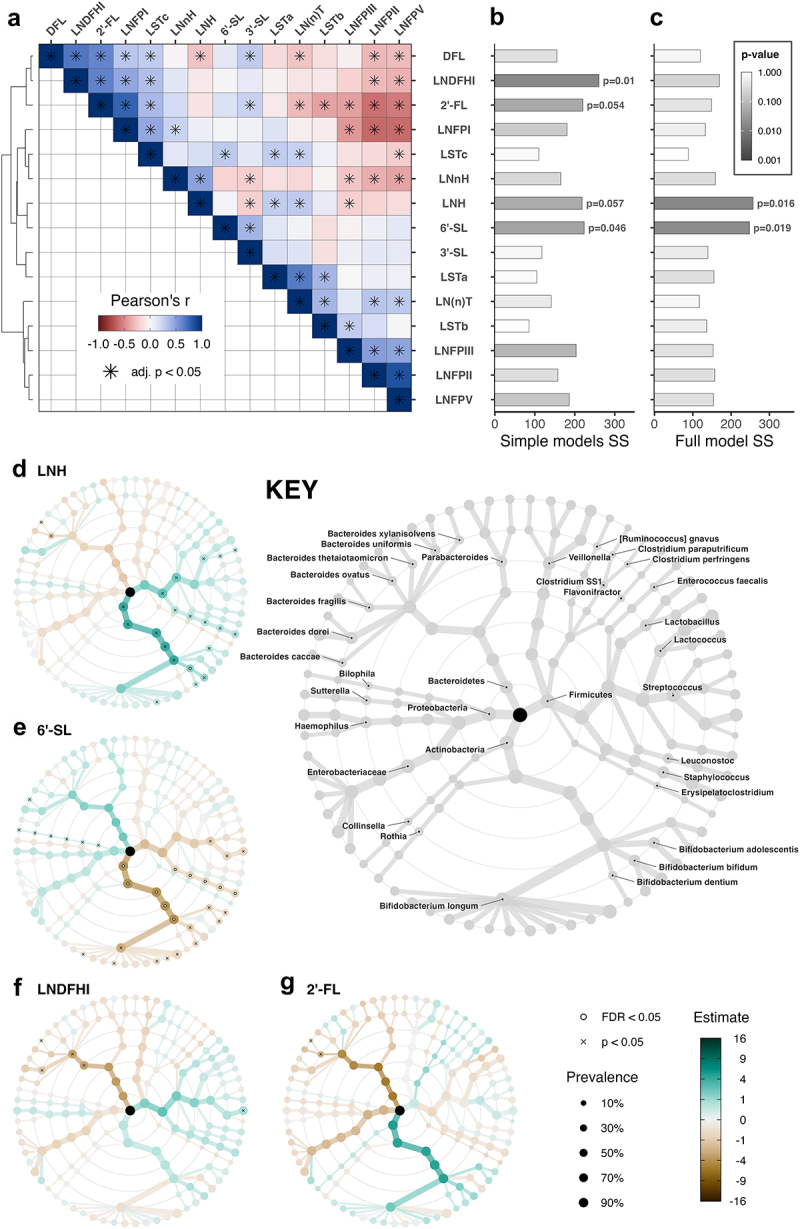
Breastmilk HMOs are highly intercorrelated, and some are associated with infant microbiota composition (N = 220).

Most major variations in HMO composition can be explained by maternal genotypes, specifically Secretor (*FUT2*) and Lewis (*FUT3*) gene variants. Non-Secretor mothers characteristically lack LNFPI and 2’-FL, whilst Lewis-negative mothers lack LNFPII, which we used to infer maternal genotype groups (Figure 3). We observed that the Secretor and Lewis statuses interacted in their influence on the resulting HMO composition. For example, the concentration of LNFPII and LNFPIII was much higher in Lewis-positive non-Secretor mothers than in Lewis-positive Secretor mothers: LNFPII mean 777 (SD = 259) vs. 246 (SD = 176) µg/mL, and LNFPIII mean 347 (SD = 129) vs. 219 (SD = 102) µg/mL. LNDFHI was highly abundant only in mothers who are Secretors and Lewis-positive. Many HMOs showed strong positive or negative correlations with other HMOs (Figure 4(a)), often with the markers of Secretor and Lewis statuses, 2’-FL and LNFPII. Synthesis of non-fucosylated neutral HMOs was not directly determined by the Secretor/Lewis genotype, but e.g. LNH was still seen at higher concentrations in the double-negative group, where the fucosylation enzymes of both *FUT2* and *FUT3* are inactive. Variations in the acidic sialylated HMOs, e.g. 3’-SL and 6’-SL, appeared uncorrelated or only weakly correlated with *FUT2*/*3* activity.

(a) Heatmaps of HMO concentration intercorrelations sorted by hierarchical clustering. Asterisks indicate FDR-adjusted significance (< .05). (b-c) Bar charts of PERMANOVA model Sums of Squares (SS), colored by unadjusted p-values for each HMO. (b) shows the results from 15 separate models and (c) from the combined model including all HMOs as covariates. (d-g) Taxonomic association trees: summary of the results of covariate-adjusted regression models of log2-transformed relative abundances, showing the strength, direction, and significance of the independent metadata-to-microbe associations modeled at all ranks from Phylum (innermost ring) to ASV (outermost ring). The color of each node represents the regression coefficients for the associations between the exposure variable and a microbial taxon. Absolute values greater than 16 are colored as ±16. Node size indicates taxon prevalence, and only taxa with prevalence of at least 5% were included in these analyses. Nodes overlaid with a white dot represent statistically significant associations after the BH correction, FDR < .05 per taxon. Crosses indicate unadjusted p < .05. KEY indicates which microbial taxon is represented at each node. See Supplementary Tables S7-9 for full results.

#### HMO-microbiota associations

Despite the substantial natural variation, we observed across breast milk HMO profiles, the influence of this variation on the breastfed infants’ gut microbiota appeared relatively modest. Neither the inferred Secretor-Lewis genotype group nor any individual HMO had a significant association with microbial alpha diversity or richness (data not shown). Moreover, we did not observe strong evidence that the inferred genotype group was associated with microbiota composition (PERMANOVA, p = .095, Supplementary Table S6).

We examined the association of each HMO with microbial composition in covariate-adjusted PERMANOVA models. Since the effect of HMOs could be expected to differ between species and strains of the same genus due to strain-level variation in HMO-degradation capacity, we calculated microbiota dissimilarities at the resolution of ASVs. In models including each HMO separately (Figure 4(b), Supplementary Table S7), we observed evidence of associations between microbiota composition and LNH (p = .057), 6’-SL (p = .046), LNDFHI (p = .017), and 2’-FL (p = .054). In a model incorporating all HMOs (Figure 4(c), Supplementary Table S8), evidence remained for associations with LNH (p = .016) and 6’-SL (p = .019).

In multivariable regression models for individual microbes (Figure 4(d), Supplementary Table S9), LNH was associated with higher relative abundances of *Bifidobacterium* (FDR = .055), especially with *B. bifidum* (FDR = .047). In contrast, higher 6’-SL concentrations were associated with lower relative abundances of *Bifidobacterium* (FDR = .015).

### Early microbiota associations with infant formula GOS supplementation

Despite variations in HMO content, breastfed infants always ingest some fermentable oligosaccharides. The same is not true for most of the 111 exclusively formula-fed infants in our study: only 18 were primarily fed formula supplemented with GOS, which became commercially available in the Netherlands shortly after the KOALA study recruitment commenced.

**Figure 5.**
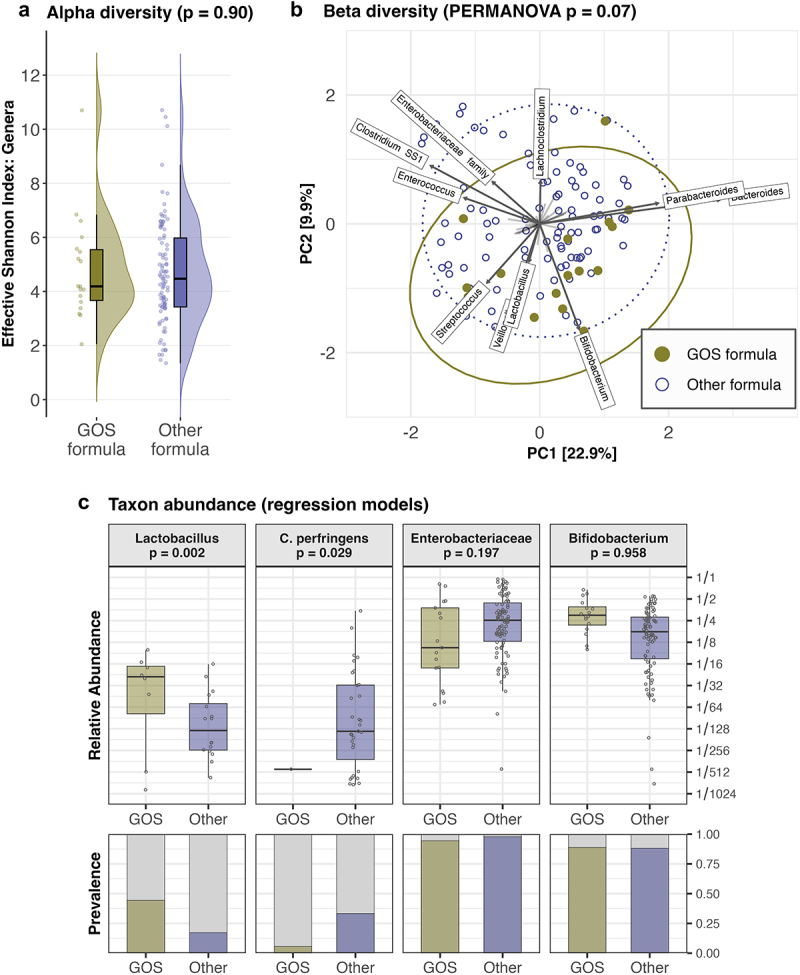
GOS supplementation and formula-fed infant microbiota composition (N = 111).

Using multivariable PERMANOVA, we tested for an independent association between GOS supplementation and microbiota composition within exclusively formula-fed infants. We identified weak evidence for an association between GOS supplementation and microbial composition (p = .072, Figure 5(b), Supplementary Table S10) and no association with alpha diversity (p = .899, Figure 5(a), data not shown).

(a) Raincloud plots showing the distribution of the effective Shannon index at genus level, grouped by the category of infant formulas. (b) PCA biplot using CLR-transformed genus-level microbial features. Labeled taxon loading vectors indicate the most major taxonomic contributions to microbiota variation along each PC axis. The 95% confidence ellipses are drawn around GOS-fed and other formula-fed infants. (c) Boxplots of taxon relative abundances (when present) on a log2-transformed scale, and bar charts of taxon prevalence. Boxplots show median and interquartile range (within hinges), with whiskers extending to samples at most 1.5 times the interquartile range from the nearest hinge. Individual samples are displayed as horizontally jittered points. p-values for all panels are derived from covariate-adjusted multivariable models (see Methods).

We observed possible microbial associations of GOS supplementation from exploratory PCA analyses (Figure 5(b)) and multivariable regression models of microbial relative abundances (Supplementary Table S11), including significant associations (p < .05, Figure 5(c)) with a higher relative abundance of *Lactobacillus* and a lower relative abundance of *Clostridium* SS1, particularly *Clostridium perfringens* (p = .03).

## Discussion

In this large-scale study, we aimed to examine the impact of determinants of the early-life gut microbiota with a special focus on antimicrobial exposures and infant feeding, including the variable prebiotic content of breast milk and infant formula.

We identified that natural variation in breast milk HMO content may influence the breastfed infant gut microbiota. In particular, LNH and 6’-SL showed significant independent associations with microbiota composition at 1 month of age, including contrasting positive and negative associations with the keystone taxon *Bifidobacterium*, respectively.

In our exploration of associations between antimicrobial exposures and the bacterial gut microbiota composition of neonates, we characterized major deviations in neonates directly exposed to antibiotics, corroborating prior findings.^5–7^ Moreover, we did not detect any neonatal bacterial microbiota associations either with maternal exposure to antibiotics during pregnancy or with neonatal exposure to oral antifungal medications.

We also confirmed the known importance of vaginal delivery in establishing the early gut microbiota,^27–32^ and detected associations between microbiota composition and homebirth, siblings, and animal exposures.

Breastfeeding showed the strongest association with microbiota composition, even at this early age, but despite its strong positive associations with most *Bifidobacterium* ASVs, not all breastfed infants were dominated by *Bifidobacterium*. Some were dominated by *Bacteroides* or *Enterobacteriaceae* instead, or showed a mixed composition. Part of this variation between breastfed infants’ microbiotas could be explained by the variation in breast milk HMO content, e.g. by the aforementioned associations of 6’-SL and LNH. However, it was somewhat surprising that the largest driver of variation in HMO profile, the genotype-associated Secretor status and Lewis grouping, did not seem to drive major variation in infant microbiota. A few smaller cohorts have previously reported weak or absent evidence for a microbial effect of the Secretor status, in early and late infancy, although their power was limited by the low prevalence of Secretor-negative mothers.^22,24,33^

We speculate, in concurrence with Laursen et al.^24^ that whilst it is likely that fucosylated HMOs are important prebiotics supporting the frequent dominance of *Bifidobacterium* in the breastfed gut, the lack of consistent associations between microbiota and fucosylated HMO profile, e.g. Secretor status, may be partly explained by the existence of substantial functional redundancy across the different fucosylated HMOs. This idea is supported by *in vitro* data demonstrating that some *Bifidobacterium* species can metabolize a broad range of HMOs and that several other taxa can metabolize various HMOs to at least some degree, including some *Bacteroides* species.^34,35^

Our cohort was large enough to include substantial numbers of non-secretor mothers and Lewis-negative mothers, including the uncommon combination of both negative phenotypes. We observed obvious interaction between the genotypic variants at the *FUT2* (Secretor) and *FUT3* (Lewis) loci in the production of fucosylated HMOs, as expected from the underlying fucosyltransferase enzyme biology,^36^ which resulted in four distinct HMO profile groups. It is not yet clear what drives interindividual HMO variation that is not attributable to Secretor or Lewis status, such as in non-fucosylated neutral HMOs and sialylated HMOs, although some reports suggested parity and birth-mode may be relevant.^37,38^

Only a few HMO structures have been subject to prior study, but already a combination of 2’-FL and LNnT has shown bifidogenic effects in formula-fed infants,^39^ and 2’-FL appeared to reduce TLR-4/5 signaling and inflammation.^40–42^ Our results provide an impetus to expand this area of research, both to include more individual candidate prebiotics, e.g. LNH, but also to better our understanding of the benefits offered by the evolved diversity of natural HMO mixtures. Additional HMOs, beyond those measured in our study, are also interesting, such as disialyllacto-N-tetraose (DSLNT), which has been associated with reduced necrotizing enterocolitis risk in preterm infants.^43^

Our observations on the effects of GOS supplementation were relatively inconclusive, likely due in part to the limited number of GOS-fed children in our formula-fed subgroup.

However, we did detect a positive association between GOS and *Lactobacillus* relative abundances and a negative association between GOS and the pathobiont *Clostridium perfringens*. Prior research with probiotics has identified that *Lactobacillus* can metabolize some GOS structures^44^ and may be able to suppress *C. perfringens* growth.^45,46^ Due to the relatively low prevalence of these two taxa in our dataset, we could not directly observe evidence of a suppression effect. Nevertheless, these findings add to other evidence of prebiotic and gastrointestinal-health benefits conferred by GOS supplementation.^17,47^

Substantial effects of direct antibiotic exposure in these term infants were apparent, despite the rarity also of this exposure. Antibiotics were associated with lower *Bacteroides* and higher *Enterobacteriaceae* and *Clostridium* SS1 species relative abundances. Many *Enterobacteriaceae* and some Clostridium species are often resistant to common antibiotics through the carriage of resistance genes,^48–50^ and spore formation may also give some Clostridia a competitive advantage after antibiotic exposure. Infections caused by multi-drug-resistant *Enterobacteriaceae* are increasingly common,^48,50^ and some Clostridia, e.g. *C. perfringens*, are common opportunistic pathogens in neonates.^51^

Two other studies have recently reported similar effects of direct antibiotic exposure on the microbiota of term neonates. One was a case–control study, which also found that a longer duration of antibiotic exposure (7 vs 2–3 days) led to more profound microbiota perturbations,^6^ and the other was a randomized trial comparing three intravenous antibiotic regimens, which found that the class of antibiotic influenced the gut microbiota impact.^52^ In combination, these results provide strong evidence for the vulnerability of the early gut microbiota to perturbation by antibiotics. This susceptibility may also implicate the infant microbiota in mediation of links between infant antibiotic exposure and atopic diseases,^15^ as the infant gut microbiota has also been linked to asthma development,^27,53^ and shown to explain part of the increased eczema risk after C-section.^54^ The microbiota associations of C-sections were similar to those of infant antibiotics, but these were independent effects, as only two of our directly antibiotic-exposed neonates were born by C-section. Trials have also demonstrated that microbiota associations of C-section persist when the direct influence of perioperative maternal antibiotics is excluded, by delaying antibiotic administration until after umbilical cord clamping.^30,55^

It has also been hypothesized that an altered infant microbiota may mediate associations between maternal exposure to antibiotics during pregnancy and increased rates of asthma and obesity in their children.^11,13,15,16^ Several animal studies have connected maternal antibiotic exposure in pregnancy with altered offspring microbiota and even topic disease,^10,12,56^ and concerns have been raised about the infant health consequences of maternal antibiotic (over-)prescription.^14^ However, no large human cohort or trial has yet demonstrated that the antibiotic regimens actually prescribed to pregnant women in clinical practice do impact the infant gut microbiota. We did not find any evidence for alteration of the gut microbiota composition of the 155 infants with antibiotic-exposed mothers, and nor did a previously smaller longitudinal cohort.^57^ Alteration of the infant microbiota may not be necessary to mediate a link between pregnancy antibiotics and child immune health, as alternative mechanisms linking maternal microbiota directly to offspring immunological development and health have also been proposed.^58^

Additionally, we found no evidence of any effect of direct infant exposure to oral antifungals, as commonly prescribed for oral thrush, on the gut bacterial microbiota. This may seem unsurprising, as the bacterial microbiota should not be directly influenced by antifungal agents. However, a recent murine study^8^ claimed a causal role for fungal pioneer species in bacterial microbiota assembly, and another found that antifungal treatment could have downstream bacterial impacts.^9^ We did not find evidence for such effects in the gut bacterial microbiota of the 37 neonates exposed to oral antifungals, but as we did not measure the mycobiome directly this remains an interesting and clinically relevant question for further study.

Due to incomplete information on the infant and maternal antibiotic treatments, we could not draw any specific conclusions regarding class, timing, duration, or indication of antibiotics. Further limitations of this study include the single timepoint of fecal and breast milk sampling and the limited resolution of taxonomic classification possible with 16S rRNA gene amplicon sequencing. In particular, the species-level classifications should be interpreted with caution, as within some clades the 16S rRNA gene V4 region targeted is highly similar or identical across multiple species. In addition, data on the breast milk microbiota composition were not available for this study, and breast milk microbes may play an important role in seeding the developing gut of breastfed infants.^24^

In conclusion, our findings from the KOALA birth cohort study provide further evidence of the vulnerability of the early gut ecosystem to disturbance by direct antibiotics but do not indicate any association of the neonatal gut bacterial microbiota with oral antifungal medication use or with maternal exposure to antibiotics during pregnancy. We suggest that an expanded study of the natural diversity of HMOs could offer insight into approaches for safeguarding the infant microbiota development. To dig deeper into individualized responses to antibiotic and prebiotic exposures, future studies should record the class, duration, and indication of antimicrobial prescriptions, and use metagenomic sequencing to characterize the community antimicrobial resistance profile and carbohydrate metabolism capacity. Ultimately, further investigation into the effects of HMOs could yield dietary strategies capable of mitigating antibiotic-induced damage, by strengthening the resilience of the gut ecosystem.

## Methods

### Study design, fecal sample, and breast milk collection

The KOALA Birth Cohort Study is a prospective birth cohort in the Netherlands, the design of which has been described in detail elsewhere.^59,60^ The KOALA study was approved by the Ethics Committee of Maastricht University Medical Center and all parents gave written informed consent. The study recruited 2843 pregnant women, using two strategies: enrollment from within a larger ongoing prospective cohort on pregnancy-related pelvic girdle pain (conventional recruitment group) and advertisements placed in locations associated with “alternative” lifestyle choices, such as organic shops, anthroposophic clinics, and Steiner schools (alternative recruitment group).

The KOALA study began collecting infant fecal samples halfway through the recruitment period (2000–2002), resulting in fecal samples being collected from 1176 one-month-old infants. As described previously,^60^ parents collected a fecal sample from a sanitary napkin placed in the infant’s diaper and mailed the collection tube to Maastricht University Medical Center the same day. We excluded samples collected <21 d or >49 d postpartum, as well as samples from premature infants (<36 weeks), twins, and infants with congenital disorders (e.g. Down’s syndrome).

On the same day as infant fecal sampling, 247 mothers also provided breast milk samples, which were collected into sterile tubes, refrigerated, and transported on ice to Maastricht University Medical Center that day.^21^ Samples were stored at −80°C in the European Biobank Maastricht, in separate lipid and aqueous fractions, as described previously.^21^ We excluded breast milk samples if the infant was fully or partially formula-fed at the time of fecal sample collection, or if <80% of the feedings from birth until sample collection were breast milk.

### Fecal microbiota profiling

Fecal DNA was isolated by repeated bead beating and column-based purification, and PCR amplification was performed using barcoded primer pairs targeting the V4 region of 16S ribosomal RNA (rRNA) genes (515 F-806 R).^21,60,61^ Eurofins Genomics, Germany, performed paired-end Illumina HiSeq sequencing of the PCR products, including mock communities and non-template controls.

We inferred amplicon sequence variants (ASVs) using NG-Tax2^62^ with default settings. We used NG-Tax2 with the SILVA-132 reference database^63^ for taxonomic annotation of ASVs and inferred a phylogenetic tree with phangorn.^64^ If an ASV could not be uniquely classified at a particular rank, they were aggregated together for the taxonomically aggregated statistical analyses under the name of the lowest classified rank, e.g. “*Enterobacteriaceae* family”. We excluded all samples with fewer than 15,000 taxonomically classified reads as low-quality samples.

See supplementary methods for further details.

### Breast milk HMO profiling

HMOs were extracted and purified from the aqueous fraction of breast milk samples as previously described,^21,65^ using Supelclean™ ENVI-Carb™ 250 mg/3 mL solid phase extraction (SPE) tubes (Sigma-Aldrich, USA). The purified HMO fraction and HMO standards were reduced to alditols using sodium borohydride, followed by SPE-based purification. Fifteen distinct HMO structures were quantified by a liquid chromatography mass spectrometry (LC-MS) device equipped with a porous graphitic carbon column (3 μm particle size, 2.1 mm × 150 mm; Hypercarb, Thermo Scientific). This included seven fucosylated structures (2′-FL, DFL, LNDFHI, LNFPI, LNFPII, LNFPIII, and LNFPV), three non-fucosylated neutral structures (LNH, LNnH, and a combination of LNT and LNnT referred to as LN(n)T) and five sialylated structures (3´-SL, 6´-SL, LSTa, LSTb, and LSTc). XCalibur4.3 (Thermo Scientific) was used for processing the LC-MS spectra into concentration estimates.

We inferred the Secretor and Lewis groups from our measurements of two fucosylated structures. Samples without 2’-FL (<10 µg/mL) were assumed to be from mothers with a nonfunctional variant of the fucosyltransferase-2 (*FUT2*) gene (non-Secretors). Similarly, the absence of LNFPII was assumed to result from a nonfunctional variant of the *FUT3* gene (Lewis-negative).

### Questionnaire data

Several questionnaires were completed by parents during pregnancy and the first months after delivery. This provided information on maternal age (years); maternal education level (higher/lower), KOALA recruitment group (conventional/alternative); gestational age (weeks); birth mode (C-section/vaginal); birthplace (home/hospital); older siblings (yes/no); furry pet or farm animal exposure (yes/no); maternal antibiotic exposure during pregnancy (yes/no); age at infant fecal sample collection (days); and milk feeding at fecal sample collection (breast/formula/mixed).

We used further information from a weekly maternal questionnaire to define an estimate of the overall proportion of feedings by breast prior to infant fecal sample collection (Breastfeeding %) and to identify infants fed primarily GOS-supplemented formulas. Antimicrobial prescriptions reported in family doctor records or parental questionnaires were used to identify neonatal oral antibiotic exposure (yes/no), as well as oral antifungal exposure (yes/no), and potential exposure to antibiotics via breast milk (yes/no).

See supplementary methods for details on missing data handling, including imputation of a limited number of values.

### Statistical analyses

All the analyses were performed with R 4.1.1.^66^ For additional details, see supplementary methods.

#### Alpha-diversity analyses

To assess the effects of antibiotics, breastfeeding, and other exposures on microbiota richness and diversity, we used multivariable linear regression models. We calculated the observed richness of genera and the Effective Shannon Index for genus-level diversity. Modeling was repeated with ASV-level richness and diversity as sensitivity analyses.

#### Beta-diversity analyses

Rare ASVs (<2.5% prevalence) were excluded before aggregating the remaining sequences into genera and applying the centered-log-ratio (CLR) transformation, as is beneficial for analysis of compositional data.^67^ We used CLR-transformed abundances in principal components analysis (PCA) scatterplots to visualize major patterns of microbiota variation.

Multivariable permutational multivariate analysis of variance (PERMANOVA) models^68^ were used to quantify associations between infant exposures and overall microbiota composition, using primarily genus-level Aitchison distances (Euclidean distance after CLR-transformation). Species and ASV features were used in alternative models, as were ecological Bray-Curtis dissimilarities and the phylogeny-aware Generalized-UniFrac distances,^69^ to explore methodological sensitivity and complementary information.

#### Taxon abundance associations

To explore associations between infant exposures and relative abundances of individual microbes, we emulated the default approach of MaAsLin2,^70^ using multivariable linear regression to model log_2_-transformed (total-sum-scaled) abundances of each microbe.

We performed this modeling for all taxonomic ranks from phylum to ASV and visualized the resulting regression coefficients and (Benjamini-Hochberg FDR-adjusted) significance level for each taxon using a tree layout, available through the microViz R package,^71^ displaying one taxonomic association tree per covariate.

#### Multivariable model covariates

We used the following minimum set of covariates for all statistical models (alpha-diversity, PERMANOVA, and individual taxon regressions): C-section birth; infant antibiotics; antibiotics during pregnancy; presence of older siblings; age at fecal sample (days); and sample depth (read count). These factors were identified as possible confounders or otherwise of interest from current literature and expertise. Models in the breastfed sub-cohort were also always statistically adjusted for the HMO measurement batch, as these measurements were completed in two rounds and so the samples differed in freezer storage duration. Numeric predictors, such as ages and HMO concentrations, were transformed to z-scores.

In models including the full cohort of 1023 infants, we were also able to include additional covariates of interest: breastfeeding (proportion); infant antifungals; maternal antibiotics whilst breastfeeding; homebirth; pet or farm animal exposure; KOALA recruitment group; gestational age (weeks); and maternal age.

## Supplementary Material

Supplemental Material

## Figures and Tables

**Table:** 

ASV	amplicon sequence variant.
CI	confidence interval.
CLR	centered log ratio.
FDR	false discovery rate.
GOS	galacto-oligosaccharides.
HMO	human milk oligosaccharide.
PC	principal component.
PERMANOVA	permutational multivariate analysis of variance.
rRNA	ribosomal ribonucleic acid.

## Data Availability

The 16S rRNA gene amplicon sequencing data for this study have been deposited in the European Nucleotide Archive (ENA) at EMBL-EBI under the accession number PRJEB52836. https://www.ebi.ac.uk/ena/browser/view/PRJEB52836
