# Correction

**DOI:** 10.1080/19490976.2023.2260604

**Published:** 2023-10-26

**Article title**: Human milk oligosaccharides, antimicrobial drugs, and the gut microbiota of term neonates: observations from the KOALA Birth Cohort Study

**Authors**: D.J.M Barnett, M.F Endika, C.E Klostermann, F Gu, C Thijs, A Nauta, H.A Schols, H Smidt, I.C.W Arts, and J Penders

**Journal**: *GUT MICROBES*

**Bibliometrics**: Volume 15, Number 1, pages 1-18

**DOI**: https://doi.org/10.1080/19490976.2022.2164152

In the analysis presented in the original version of this article, an error was introduced when transforming microbiota abundance data before fitting regression models for the individual taxa. The authors intended, as correctly stated in the methods, to aggregate microbiota abundance data at the specified taxonomic ranks prior to log transformation. Instead, the data were aggregated after applying the log transformation.

This mistake has been corrected in the current version of this article.

The *p* and *FDR* values have been corrected in the main text, and corrected statistical results are presented in Figures 2, 4, and 5, and in the supplementary tables S5, S9, and S11. The pattern of results remain largely similar to the original version, and the conclusions are almost entirely unchanged. The notable exception to this is that the associations of the HMOs LNH and 6’-SL with *Bifidobacterium* are both not statistically significant in the corrected analyses. The clause mentioning these associations has been removed from the abstract.

**Figure 2:**
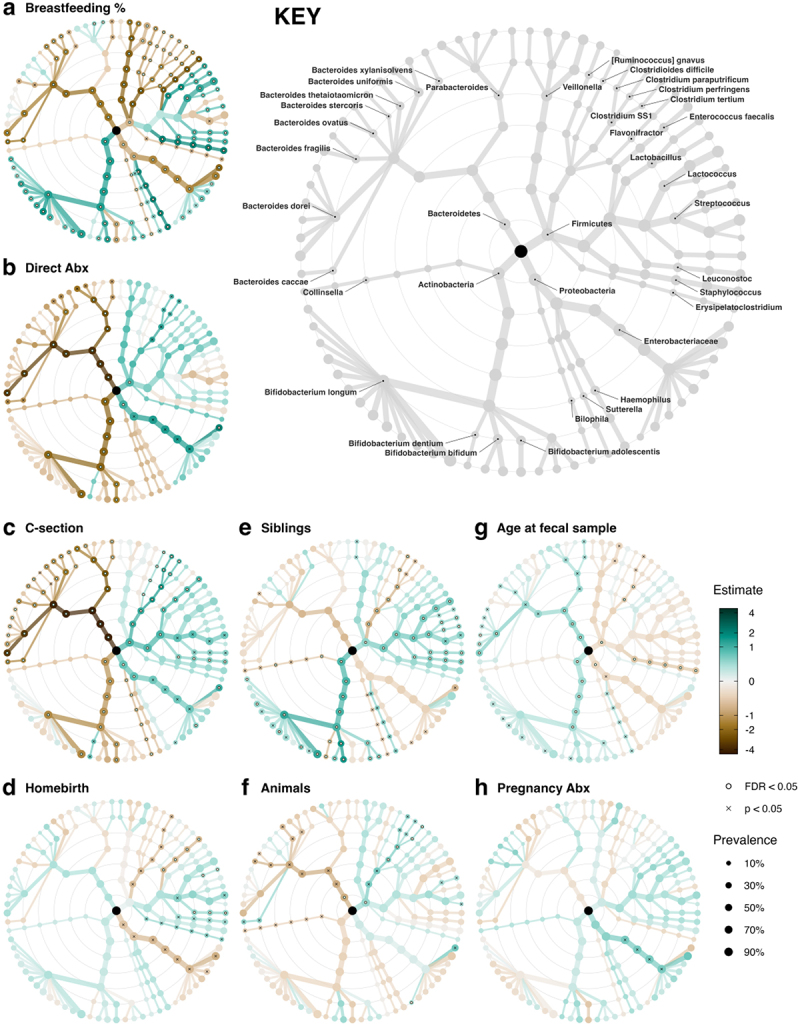

**Figure 4:**
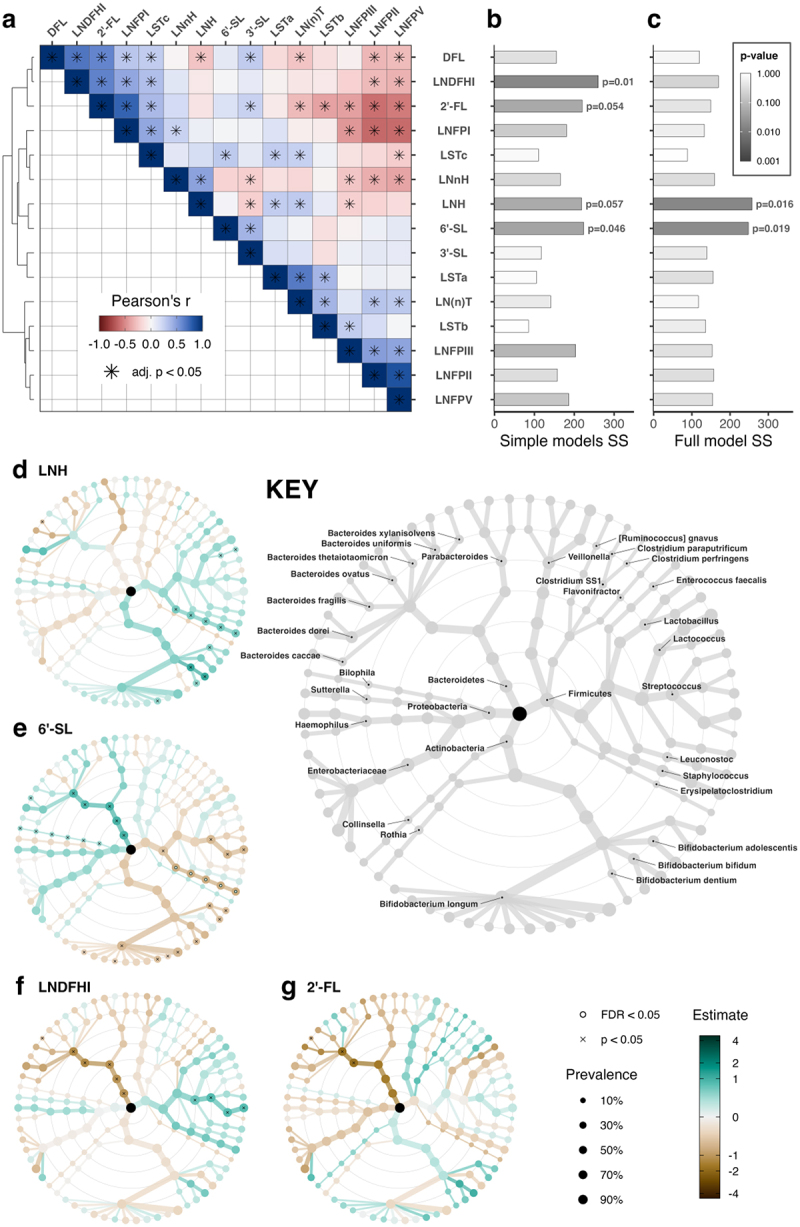

**Figure 5:**
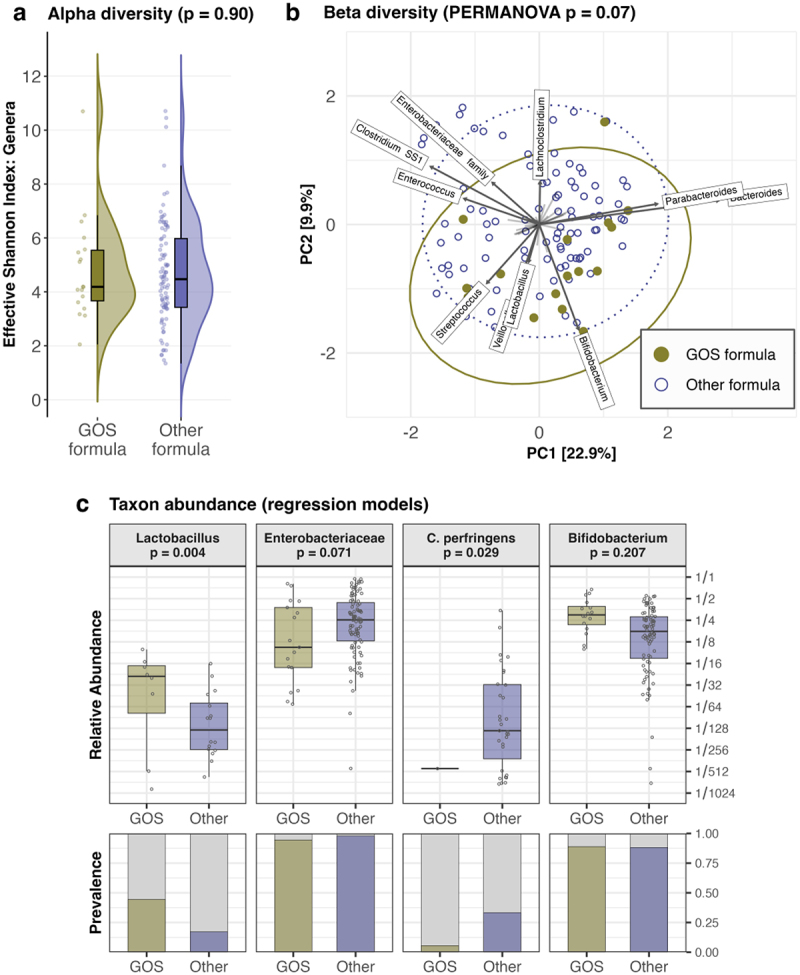

The authors apologize for any inconvenience caused.

The corrected figures are given below:

