# Supplementary material for: Human milk oligosaccharides, antimicrobial drugs, and the gut microbiota of term neonates: observations from the KOALA birth cohort study
Source: Gut Microbes. 2023 Jan 8;15(1):2164152. doi: 10.1080/19490976.2022.2164152 (PMC9833409; doi:10.1080/19490976.2022.2164152)
Supplement: Supplemental Material [file KGMI_A_2164152_SM9501.zip › S1 Population characteristics.docx]

**Supplementary Table S1: Population Characteristics**

|  | **Number (%)** | **Mean (SD)** | **N-missing** |
| --- | --- | --- | --- |
| Total | 1023 |  |  |
| Direct infant antibiotics | 23 (2.2) |  |  |
| Infant oral antifungals | 37 (3.6) |  |  |
| Antibiotics via breastmilk | 17 (1.7) |  |  |
| Pregnancy antibiotics | 155 (15.2) |  | 3 |
| C-section birth | 114 (11.2) |  | 5 |
| Homebirth | 476 (46.8) |  | 5 |
| Pet or farm animal exposure | 421 (42.0) |  | 20 |
| Older Siblings | 616 (60.2) |  |  |
| Alternative KOALA recruitment group | 323 (31.6) |  |  |
| Maternal higher education | 571 (55.8) |  |  |
| Breastfeeding (% of feeding) |  | 77.4 (35.7) | 70 |
| Gestational age at birth (weeks) |  | 39.6 (1.3) | 4 |
| Age at faecal sample (days) |  | 32.1 (4.0) |  |
| Maternal age at birth (years) |  | 32.7 (3.8) |  |
| Fecal sample sequencing depth (reads) |  | 170776 (81570) |  |
