# Supplementary material for: Human milk oligosaccharides, antimicrobial drugs, and the gut microbiota of term neonates: observations from the KOALA birth cohort study
Source: Gut Microbes. 2023 Jan 8;15(1):2164152. doi: 10.1080/19490976.2022.2164152 (PMC9833409; doi:10.1080/19490976.2022.2164152)
Supplement: Supplemental Material [file KGMI_A_2164152_SM9501.zip › Supplementary Figure 1.docx]

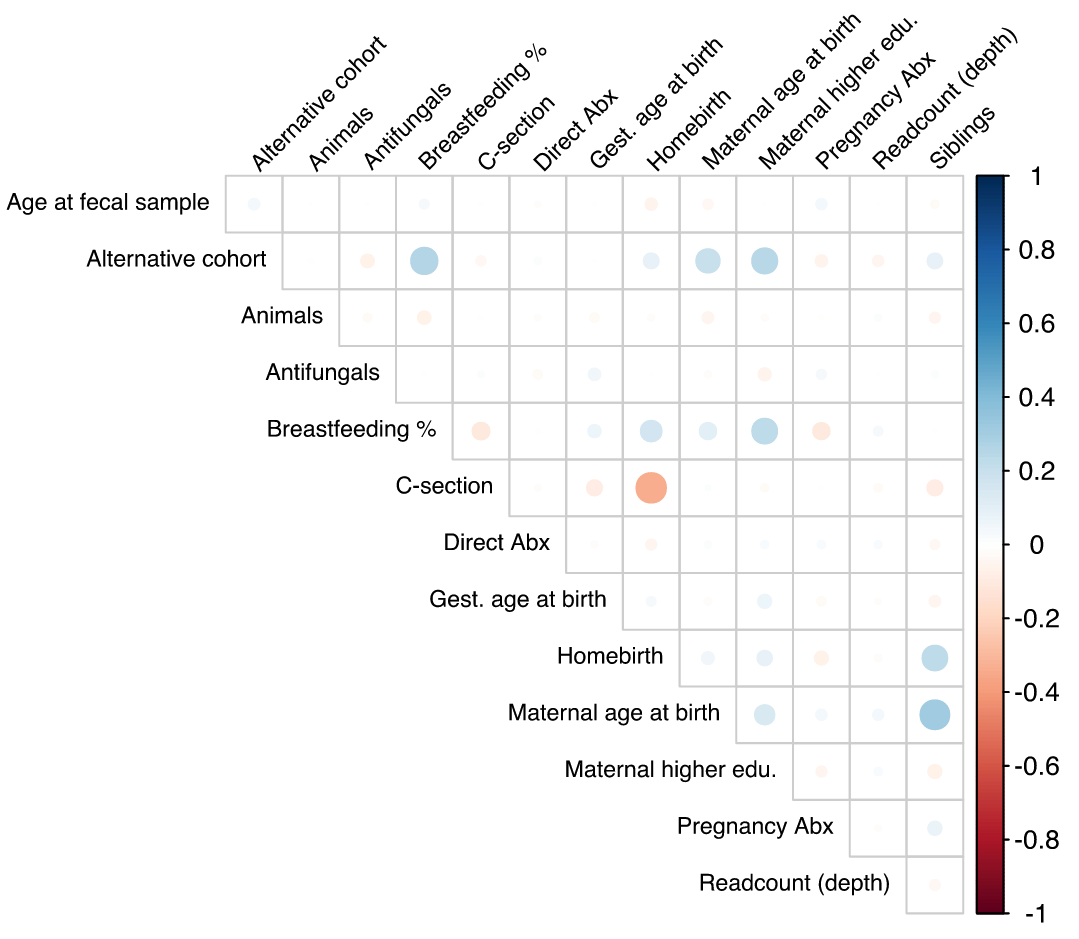


**Supplementary Figure 1. Little correlation between most model covariates (N = 1023).**

Heatmap of Pearson correlations coefficients between pairs of exposures used as predictors in the main covariate-adjusted statistical models of neonatal microbiota composition (PERMANOVA), alpha-diversity, and taxon relative abundance (regression). Point size represents magnitude of correlation.
