# Supplementary material for: Human milk oligosaccharides, antimicrobial drugs, and the gut microbiota of term neonates: observations from the KOALA birth cohort study
Source: Gut Microbes. 2023 Jan 8;15(1):2164152. doi: 10.1080/19490976.2022.2164152 (PMC9833409; doi:10.1080/19490976.2022.2164152)
Supplement: Supplemental Material [file KGMI_A_2164152_SM9501.zip › Supplementary_Methods.docx]

# Supplementary methods

This document contains additional details, supporting the Methods section within the manuscript.

### Breastmilk collection

Breastmilk samples were collected into sterile tubes (Cellstar PP-test tubes, Kremsmünster, Austria). The samples were centrifuged (400 × g, 12 min, no brake, 4 °C) and the lipid and aqueous fractions were separated and stored in plastic vials (Sarstedt, Nümbrecht, Germany) at -80 °C. The remaining debris was not used to avoid contamination with cell fragments.

### Faecal microbiota data generation

#### Laboratory methods

Column-based purification of extracted DNA was performed using the QIAamp DNA stool mini kit (Qiagen, Hilden, Germany) according to the manufacturer's instructions. PCR amplification was performed in triplicate, using 25 cycles for reactions containing 1.4 – 20 ng DNA template or 30 cycles for <1.4 ng DNA template.

#### Sequence data processing

We inferred amplicon sequence variants from the sequence data using the NG-Tax2 pipeline with default settings, and trimming the forward and reverse reads to the length of 80 bases. Any reads assigned to the genera *Ralstonia* or *Cupriavidus* were removed from the dataset prior to statistical analyses, as these are known to commonly contaminate lab kits and were consistently detected in our negative control data.

### Questionnaire data detail

We obtained detailed information on infant feeding pattern from a maternal questionnaire completed for the first 12 weeks after birth, which included weekly reporting of feeding mode (breast/formula/mixed) and the name(s) of any formula product(s) given. This information was used to define an estimate of the proportion of feeding by breast prior to the moment of infant faecal sample collection (counting breastfeeding as 1, mixed feeding as 0.5, and formula feeding as 0 at each weekly time-point and taking the mean). We also used this questionnaire to identify infants fed formula products known to contain galacto-oligosaccharides (GOS).

We identified reports of antimicrobial usage from a combination of family doctor records, weekly questionnaires and questionnaires completed upon fecal sample and breastmilk collection. A relevant report on any one of these sources was used to classify cases of neonatal oral antibiotic exposure prior to faecal sample collection (yes/no) as well as oral antifungal exposure (yes/no) and potential exposure to antibiotics via breastmilk (yes/no).

### Missing data handling

Complete data were available for the majority of model covariates, however as the weekly feeding questionnaire was not completed by all participants, data on the proportion of infant feeding by breast was missing for 73 of the 1023 participants. These missings were imputed with predicted values from linear regression models, which included informative auxiliary variables from other KOALA questionnaires: infant feeding at time of faecal sample, as well as reported age of breastfeeding cessation and age at first formula feeding, when available.

Additionally, for 21 participants neonatal animal exposure status could not be ascertained from postnatal questionnaires, so we imputed this using predictions from a random forest model (R package randomForest) based on auxiliary variables about family pet ownership reported during pregnancy.

Birth mode and place data were missing for five participants, and were imputed with random forest model predictions based on other covariates in this study. Lastly, three further variables each had fewer than five missing values. These were maternal antibiotic exposure during pregnancy, age at one-month faecal sample, and gestational age, and were each respectively imputed with mode, median, and median values.

### Statistical methods

#### Exclusion of rare taxa

We excluded rare taxa by applying a 2.5% minimum prevalence threshold to ASV-level microbial data. At the same time we excluded sequences with very few reads, by removing ASVs that did not represent at least 1 in every 10,000 reads, when totalled across all samples. This filtering was done for all analyses except alpha diversity calculation, for which no filtering was applied, and the multivariable linear regression modelling of the log2-transformed relative abundances of individual taxa, for which a stricter prevalence threshold of 5% was applied, with a high detection threshold, requiring at least 500 reads to count as present in a sample. This latter filtering criterion helped to remove very low abundance variants (likely erroneous) that often co-occurred with highly-abundant *Bifidobacterium* or *Enterobacteriaceae* sequences.

#### Ordination visualisations

Ordinations were performed using the vegan R package (Oksanen et al., 2020), and visualised with microViz R package (Barnett et al., 2021) and the ggplot2 R package (Wickham, 2016). PCA loading scores for each taxon were overlaid on the same coordinates with a consistent arbitrary scaling, to provide an indication of the relative contribution of taxonomic features to the PCA dimensions shown.

#### PERMANOVA

Multivariable permutational multivariate analysis of variance (PERMANOVA) models were run with 9999 permutations, using the adonis2 function from the R package vegan (Oksanen et al., 2020). Where generalised UniFrac distances were used, we applied the default alpha setting of 0.5.

#### Log transformations pseudocounts

We applied centred-log-ratio transformations (for PCA and PERMANOVA) using the R package microbiome (Lahti and Shetty, 2012), which prepares the dataset for log transformation by first adding a pseudocount to remove zeros, equivalent to half of the dataset’s minimum proportional abundance value. Prior to log2 transformations, for taxon abundance regression models, we added a pseudocount in the same way.

#### Heatmap clustering

Where hierarchical clustering was applied to sort heatmap data, the Ward method was used with further optimisation by optimal leaf ordering, as implemented in the R package seriation.
